# Validating the Social Vulnerability Index for alternative geographies in the United States to explore trends in social determinants of health over time and geographic location

**DOI:** 10.3389/fpubh.2025.1547946

**Published:** 2025-03-04

**Authors:** Carmen D. Ng, Pluto Zhang, Stacey Kowal

**Affiliations:** Genentech, Inc., South San Francisco, CA, United States

**Keywords:** Social Vulnerability Index, social determinants of health, real-world data, health equity, geography

## Abstract

**Objective:**

To create county-, 5-digit ZIP code (ZIP-5)–, and 3-digit ZIP code (ZIP-3)–level datasets of the Social Vulnerability Index (SVI) and its components for 2016–2022 to validate the methodology beyond county level, explore trends in SVI over time and space, and demonstrate its usage in an enrichment exercise with health plan claims.

**Materials and methods:**

The SVI consolidates 16 structural, economic, and demographic variables from the American Community Survey (ACS) into 4 themes: socioeconomic status, household characteristics, racial and ethnic minority status, and housing type and transportation. ACS estimates of the 16 variables for 2016–2022 were extracted for counties and ZIP code tabulation areas and for ZIP code geographies, crosswalked to ZIP-5, and aggregated to ZIP-3. Areas received a percentile ranking (range, 0–1) for SVI and each variable and composite theme, with higher values indicating greater social vulnerability.

**Results:**

SVI estimates were produced for up to 3,143 counties, 32,243 ZIP-5s, and 886 ZIP-3s. SDoH trends across the US were largely consistent from 2016 to 2022 despite slight local changes over time. SVI varied across regions, with generally higher vulnerability in the South and lower vulnerability in the North and Northeast. When linked with health plan claims data, higher SVI (i.e., higher vulnerability) was associated with greater comorbidity burden.

**Conclusion:**

SVI can be estimated at the ZIP-3 and ZIP-5 levels to provide area-level context, allowing for more routine integration of socioeconomic and health equity–related concepts into health claims and other datasets.

## 1 Introduction

Disparities in health outcomes in the US are prevalent across a variety of dimensions—including but not limited to race and ethnicity, socioeconomic status, and location. While causal pathways underpinning existing health disparities are complicated, it is clear that they are driven by numerous structural and individual aspects of social disadvantage, including structural racism and bias, socioeconomic status, race and ethnicity, gender, geographic location, disability, and others (1, 2). These factors and other place-based root causes affect local residents’ access to safe water, healthy food, decent housing, high-quality health care, and strong educational opportunities (3, 4).

Real-world evidence demonstrates that these disparities exist over a variety of key healthcare outcomes, including mortality, morbidity, access to healthcare resources, and ability to participate in clinical trials (5–7). The intersection of individual sociodemographic characteristics, place, and health has been studied by many researchers, with remarkably consistent conclusions, showing that disadvantaged groups tend to have worse health than more advantaged groups across a range of outcomes (3, 4).

A number of parties have paid increased attention to health disparities in the US, including governments and health policymakers (8). Addressing health disparities and advancing health equity are priorities for the Centers for Medicare and Medicaid Services, the largest provider of US health insurance, as well as other governmental agencies such as the Centers for Disease Control and Prevention (CDC) and the US Food and Drug Administration (9, 10). For many health equity efforts, the ultimate goal is to identify and remedy the systemic barriers that are causing these disparities, so all people have a fair and just opportunity to attain the highest level of health (11). Real-world evidence can play a critical role in identifying these systemic barriers, as there is an increasing emphasis on using real-world data (RWD) to understand and analyze health equity. However, the causal pathways and interactions across many intertwined factors at different levels (e.g., individual vs. neighborhood) are complex and difficult to untangle (12). To develop tangible ideas about policy action and practice innovation, a more comprehensive picture of existing inequalities and how individual characteristics intersect with socioeconomic opportunities at the local level is needed, along with a discrete implementation strategy for focusing the attention of key decision-makers on the disparities in those localities. To ensure we can better address questions on equity, data enrichment efforts have aimed to gain a deeper understanding of patients and their experiences with broader social and material conditions impacting health (6).

Social determinants of health (SDoH) are the conditions in which people spend their time that affect a wide range of health, functioning, and quality-of-life outcomes and risks (13–15). Although individual-level characteristics (including age, gender, race and ethnicity, education, and income) are important, they are not substitutes for SDoH, which provide contextual insight into the environments and social constructs in which people interact on a day-to-day basis (13–15). For example, there are likely differences between a high-earner living in a high-income area and someone making the same income in a low-income area, such as the types of services they have access to in their neighborhood and their lived experiences (13–15). Many structural- and system-level factors play a large role in shaping health outcomes across geographies, including factors such as access to healthcare facilities, educational systems and opportunities, and levels of structural racism (16, 17).

SDoH are a key focus of Healthy People 2030, and their 5 domains of SDoH are education access and quality (e.g., percentage of population with a high school diploma), healthcare access and quality (e.g., health insurance enrollment rate), economic stability (e.g., median household income), neighborhood and built environment (e.g., percentage of households that are in mobile homes), and social and community context (e.g., crime rate) (18). These SDoH concepts can be aggregated and combined with additional local data in area-level indices, which distill the characteristics of a geographic area into a single holistic metric and allow for easy interpretation and visualization (19). Stakeholders can use these indices to identify geographic areas and patient populations at particular risk of specific health conditions or of morbidity and mortality related to those conditions, target areas of unmet need, and assess the benefit of public health and policy interventions to break down systemic barriers and improve health outcomes (20, 21).

Numerous area-level indices exist in the United States to measure area-level socioeconomic variation and assess community needs (16). The Social Vulnerability Index (SVI) is one such index that was constructed by the CDC, with an initial focus on disaster preparedness. The CDC produces SVI estimates for census tracts and counties every other year (22). It is a freely available measure with transparent documentation on methods and data sources that is increasingly used in healthcare research to explore a range of questions on health disparities (23). Of note, in recent years, the SVI has been used to assess disparities in COVID-19 burden and outcomes and was subsequently used to support equitable rollout of COVID-19 vaccination policies (20, 24, 25). Further, recent work to map US health disparities across race, ethnicity, and geography leveraged the SVI to capture geographic differences in health (26). However, use of the SVI has been limited in RWD studies that use datasets like patient-level insurance claims or electronic health records.

Datasets commonly used for research on health outcomes may not contain information about a patient’s geography of residence at the level that a researcher would ideally have. As an example, while the county may be a useful administrative and political unit at the level of granularity that a researcher is hoping for, the risk of patient re-identification often results in location data that are provided at a higher geographic aggregation, such as the 3-digit ZIP code area (ZIP-3, which is the first 3 digits of a 5-digit ZIP-code area [ZIP-5]), or state or region level. Importantly, estimates from the annually administered American Community Survey (ACS), from which the SVI is derived, exist for different geographic units, including census tract and county, but also state, metropolitan/micropolitan statistical area, school district, ZIP code tabulation area (ZCTA), etc. (27). This is noteworthy because different geographic units could be useful for different contexts, with considerations such as the granularity desired for analysis (e.g., census tracts nest within counties, which nest within states) and the purpose of the analysis (e.g., zip code–based geographic units are based on US Postal Service [USPS] delineations, whereas state boundaries are political) (28). Furthermore, the methodology used to construct the SVI can be applied to data for other geographic areas. SVI estimates produced for geographic units beyond census tracts or counties can then be linked with these other datasets to provide a more robust social context for patients.

Health equity can be better addressed in the US if we can develop a more nuanced understanding of health disparities, so there is a demonstrated need for more robust integration of health and deprivation measures, like the SVI, with patient-level data in a privacy-compliant manner. Greater availability of SVI and its component measures across various geographic units can allow for linking of such information into a broader set of RWD sources. The objective of this study was to establish a repeatable methodology to generate SVI for counties, ZIP-5s, and ZIP-3s in the US using CDC documentation and by applying additional methodological considerations, including imputation and geographic crosswalks for alternative geographies. Further, the study demonstrates how SVI data at the ZIP-3 level can be integrated into a health claims analysis to add additional information on drivers of disease burden. Ultimately, the mapping framework detailed in this study seeks to promote more consistent real-world evidence generation in the health disparities space.

## 2 Materials and methods

### 2.1 Data source

ACS estimates from 2016–2022 were used to construct SVI (29). The ACS is an annual demographics survey program conducted by the US Census Bureau with an aim to develop estimates of social, economic, demographic, and housing characteristics across the country. The ACS covers approximately 3.5 million households and provides reliable estimates of population demographics and socioeconomic variables in the US. There are several versions of the ACS (e.g., 1-year estimates, 5-year estimates), and 5-year estimates were used for this analysis, as they have no restrictions based on population size (i.e., sparsely populated areas are not suppressed) and are the most representative at our geographic levels of interest (29). As a result, the 2022 data release refers to data from 2018–2022; similarly, the 2016 data release refers to data from 2012–2016. We used 7 years (2016–2022) of ACS 5-year estimates. This analysis used 2016 as the starting year because this was the first full year of *International Classification of Diseases, Tenth Revision, Clinical Modification* (*ICD-10*) diagnostic coding and 2022 as the ending year because this was the latest release of the ACS. ACS estimates are aggregated to various geographic locations, including county and ZCTA (30). ZIP codes are a trademark of the USPS and are used to coordinate mail handling and delivery. While ZIP codes are not an ACS-supported geography, the US Census Bureau created the ZCTA as a means to crosswalk to ZIP code (31).

### 2.2 Social Vulnerability Index

SVI indicates the relative vulnerability of geographic areas of interest across the US (32). SVI ranks geographic locations based on 16 SDoH variables (below 150% poverty, unemployed, housing cost burden, no high school diploma, no health insurance, aged ≥65 years, aged ≤17 years, civilian with a disability, single-parent household, English language proficiency, race and ethnicity, multi-unit structures, mobile home, crowding, no vehicle, and group quarters) and further groups these variables into 4 SDoH themes (socioeconomic status, household characteristics, racial and ethnic minority status, and housing type and transportation) (Supplementary Figure 1). Each geographic region receives a ranking for each of the variables and themes, as well as an overall ranking (i.e., SVI itself). SVI scores range from 0 to 1, with a higher score indicating that an area is more vulnerable. The CDC produces these estimates at the census-tract and county level, but the same methodology can be used to create these estimates for other geographic areas.

### 2.3 Methodology to construct SVI

**Figure 1:**
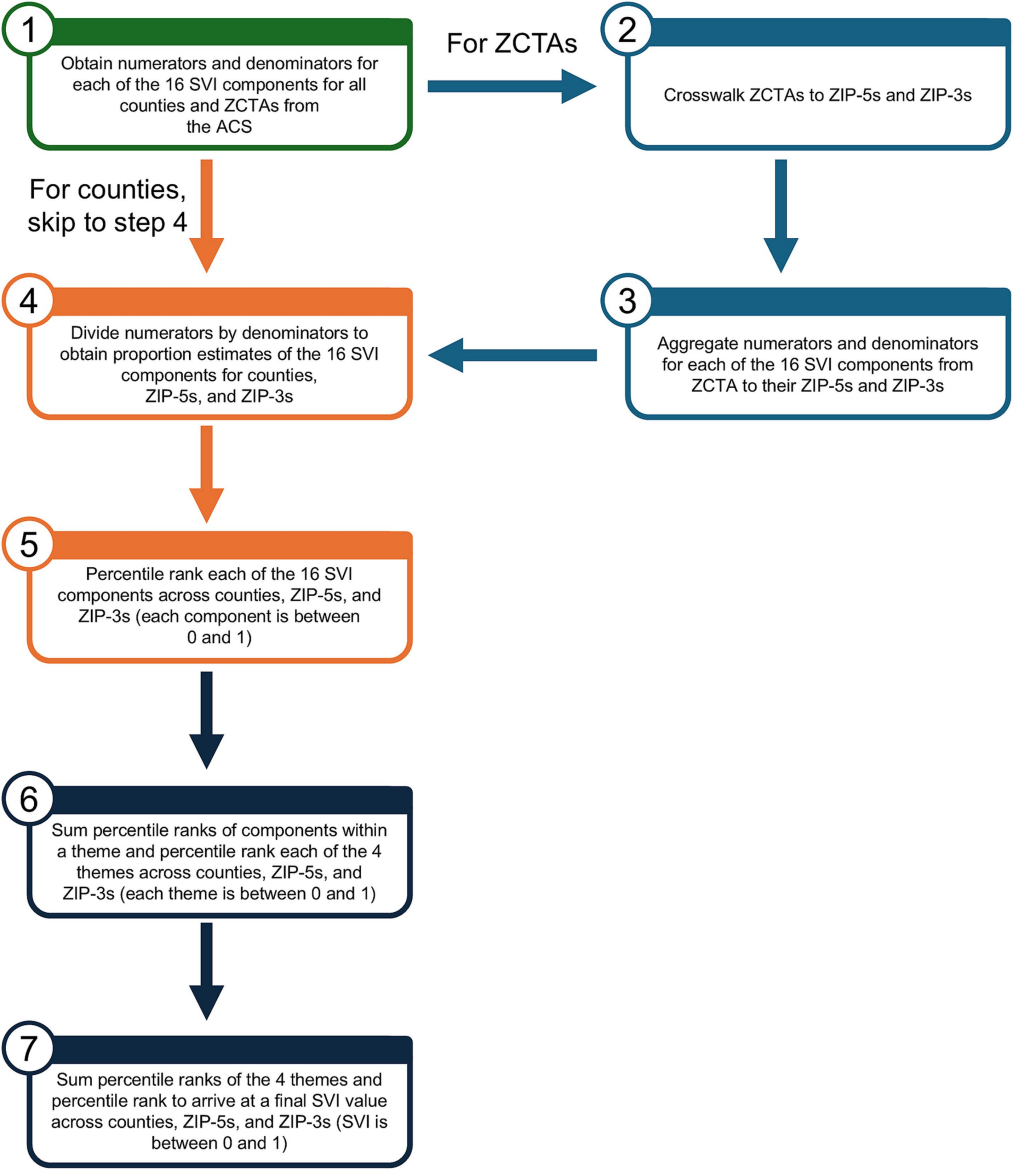
Flow chart for construction of SVI. ACS, American Community Survey; SVI, Social Vulnerability Index; ZCTA, ZIP code tabulation area; ZIP-3, 3-digit ZIP code; ZIP-5, 5-digit ZIP code.

The methodology used to construct SVI extracted data on 16 individual SDoH variables combined these 16 variables into 4 relevant SDoH themes and then combined these 4 themes into a single holistic metric for a given geographic area (Figure 1). At a high level, the combination was done through a series of percentile ranks across geographic units and sums. Each of the 16 SDoH variables was expressed as a percentage (e.g., percentage of people in a geography below 150% of the poverty line). The variables needed to form the numerators and denominators of these percentages were obtained from the ACS 5-year estimates at the county and ZCTA level (30). For ZIP-5 and ZIP-3 geographies, ZCTAs were matched to ZIP-5 using a geographic crosswalk; ZIP-5s were then truncated to ZIP-3s (i.e., the first 3 digits of the ZIP-5) (33). The ZCTA numerator (e.g., people in a geographic unit below 150% of the poverty line) and denominator (e.g., people residing in the geographic unit) values were aggregated to the ZIP-5 and ZIP-3 level to obtain totals representative of the geography of interest. With all numerators and denominators now representing the correct geography, the percentages of each of the 16 variables were calculated for counties, ZIP-5s, and ZIP-3s by dividing each numerator by the corresponding denominator. Each of the 16 variables were then ranked by percentile across all geographies, resulting in a number between 0 and 1 for each geographic area and SDoH variable, which aligns with the scale used to report the SVI (ranging from 0 [least vulnerable] to 1 [most vulnerable]). The percentile ranks of the SDoH variables in each theme were summed and re-ranked (aligning to the same 0 to 1 scale), and the percentile ranks of the themes were summed and re-ranked to form the SVI. By design, the same interpretation for SVI, theme percentile ranks, component percentile ranks, and raw component percentages can be used regardless of the geographic unit of choice.

A representative example of the workflow from below 150% poverty to SVI at the ZIP-3 level is shown in Supplementary Figure 2. The proportion of missing values for any given variable was small (~0.1%). Where applicable, we used a variation on mean imputation. For more details on the geographic crosswalk and use of imputation, see the Supplementary material.

### 2.4 Validation using CDC estimates

The CDC has released estimates of SVI for census tracts and counties biennially since 2014. Given the availability of official SVI data, we performed a validation check of the county-level data generated using our methodology compared with the CDC official estimates. The 2020 county results should be the same, as this is the methodology to which we anchored. For other years, our county results should be visually similar to, but not necessarily the same as, the census-tract and county SVI values produced by the CDC, given that there are slight changes in methodology between versions.

### 2.5 Demonstration of utility: linking claims data with ZIP-3 SVI

We sought to link our newly generated estimates of SVI to an RWD asset to explore their utility in an integration exercise. IQVIA PharMetrics Plus is a health plan claims database comprising fully adjudicated medical and pharmacy claims. It contains some basic demographic information (e.g., sex and year of birth) and has a variable for a patient’s ZIP-3 (if the ZIP-3 has at least 20,000 people), but otherwise SDoH information is not directly captured. The database includes diagnoses that can be tied to patient outcomes and mortality risk through other indices, such as the Charlson Comorbidity Index (CCI).

*ICD-10* diagnosis codes in claims provide insight into patient health. The CCI (34) is a widely used scoring system for comorbidities and predicts mortality in patients who may have several concurrent conditions. The CCI includes a range of conditions, such as diabetes, HIV and AIDS, malignancy, and dementia. A score of 0 means that a patient has no comorbidities, whereas higher values suggest a higher predicted mortality rate. In this way, the CCI can be considered a measure of sickness or disease severity. *ICD-10* codes can be used to identify the presence of the comorbidities included in the CCI.

We evaluated the relationship between CCI and SVI in the IQVIA PharMetrics Plus database of adult patients aged 18–64 years in California who were continuously enrolled in the year 2021. This exercise was done for the year 2021 to demonstrate its feasibility for years in which the CDC does not release SVI estimates (nevertheless, the CDC does not publish estimates at the ZIP-3 level in any year), but any year or even multiple years could be used. Similarly, any geography could have been chosen (e.g., a different state, a region, the whole US) as long as it contains multiple geographic units of interest to create groupings. We ordered the available ZIP-3s in California by SVI and split them into quintiles, placing each patient into 1 of 5 SVI groups; quintile 1 represents the least vulnerable group, and quintile 5 represents the most vulnerable group. The use of quintiles to estimate disparities in population groups is an approach with growing popularity and allows for comparison between the best- and worst-off populations in an area (26, 35), although other groupings (e.g., quartiles, deciles) could also be used. For this exercise, patients were grouped by age to ensure that potential differences in age distributions across geographic areas did not affect the results.

## 3 Results

### 3.1 Visualizing ZIP-3–level SVI and SDoH variables

**Figure 2:**
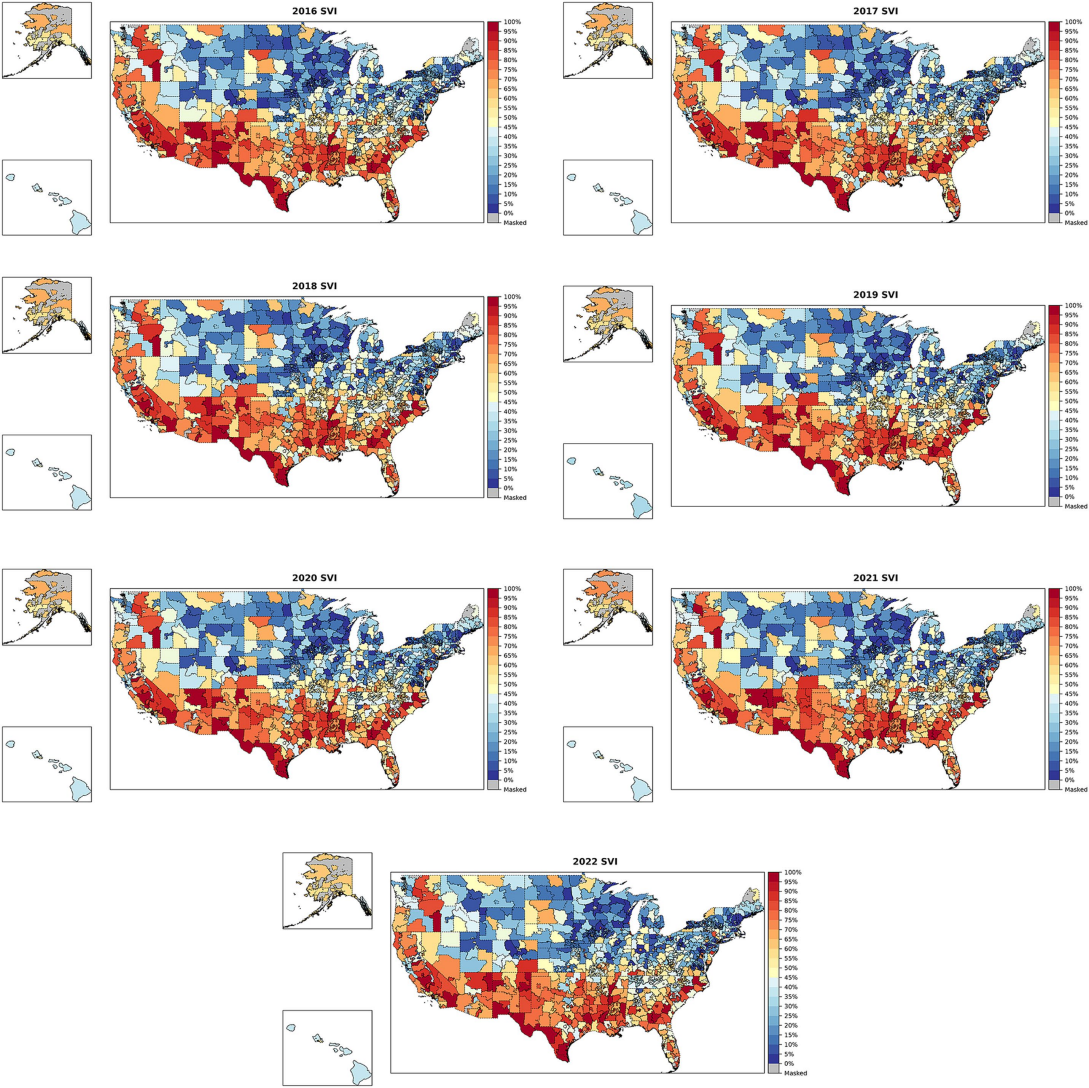
SVI across ZIP-3s in the US from 2016–2022. SVI, Social Vulnerability Index; ZIP-3, 3-digit ZIP code. Blue indicates that the ZIP-3’s SVI is closer to 0 (lower social vulnerability), while red indicates that the ZIP-3 has an SVI closer to 1 (greater social vulnerability). Gray indicates that data do not exist for a ZIP-3 because no people live in that area (e.g., Washington, DC, or post office boxes).

SVI was derived for up to 3,143 counties, 32,243 ZIP-5s, and 886 ZIP-3s from 2016–2022. The 886 ZIP-3s remained consistent across the 7 years. Choropleth maps, which are a type of thematic map used to represent data through shading or coloring of predefined geographic areas, of SVI from 2016–2022 for these 886 ZIP-3s are shown in Figure 2. SVI trends across the US from 2016–2022 were relatively consistent, despite some slight local changes over time. That is, ZIP-3s with high SVI one year tend to also have high SVI in another year, and similarly ZIP-3s with low SVI one year tend to also have low SVI in another year. SVI varied drastically over different regions of the US, with generally higher vulnerability (closer to 1) in the South and lower vulnerability (closer to zero) in the North and Northeast.

**Figure 3:**
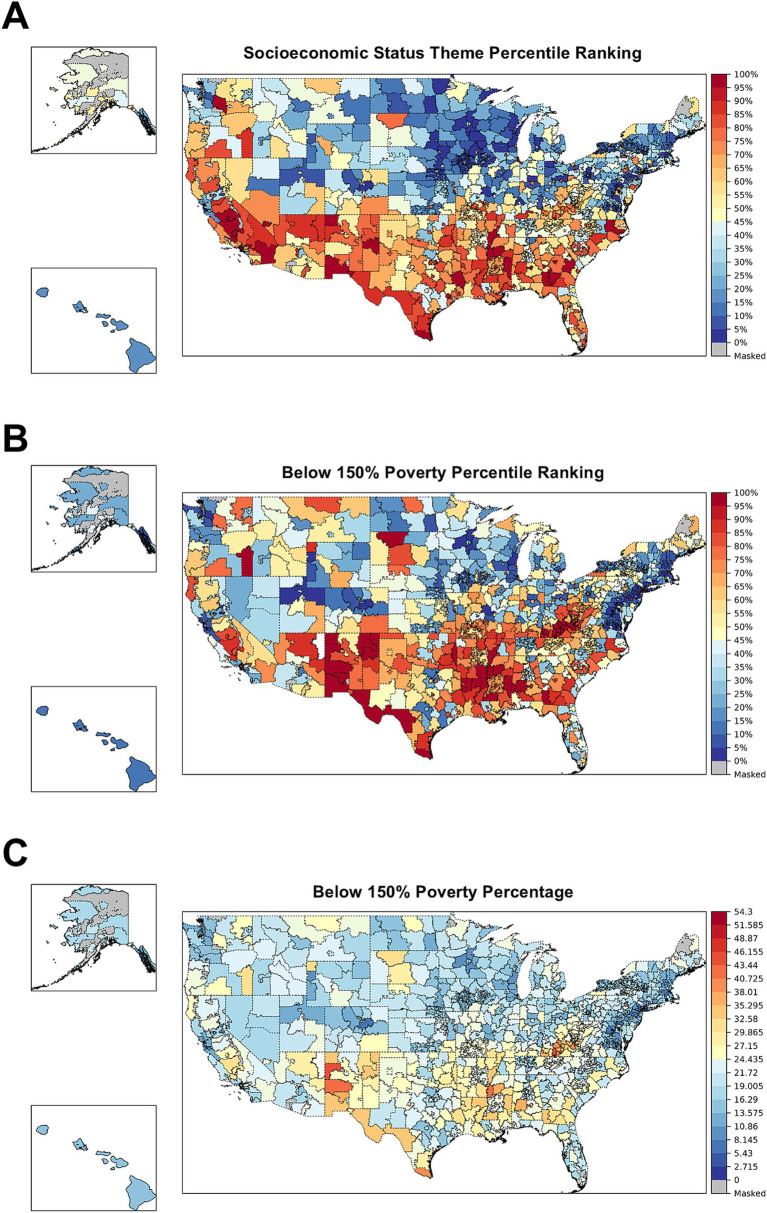
Socioeconomic status theme percentile ranking **(A)**, below 150% poverty percentile ranking **(B)**, and below 150% poverty percentage **(C)** across ZIP-3s in the US in 2022. SVI, Social Vulnerability Index; ZIP-3, 3-digit ZIP code. Blue indicates that the ZIP-3’s SVI is closer to 0 (lower social vulnerability), while red indicates that the ZIP-3 has an SVI closer to 1 (greater social vulnerability). Gray indicates that data do not exist for a ZIP-3 because no people live in that area (e.g., Washington, DC, or post office boxes).

Information on specific themes and SVI variables can also be leveraged to better understand which SDoH domains drove SVI in specific regions. Choropleth maps for the socioeconomic status theme percentile ranking, below 150% poverty percentile ranking, and below 150% poverty percentage for 2022 are shown in Figure 3. The choropleth maps for the socioeconomic status theme percentile ranking and below 150% poverty percentile ranking (the top 2 panels) were similar to the overall SVI map for 2022, with values for the geographic units stretched to be uniform over 0 to 1 by design. The choropleth map for the actual below 150% poverty percentage in the bottom panel shows a maximum value of 54%, with most ZIP-3s generally having a value of <30%. The panels showing the percentile rankings help elucidate important differences across geographic units, whereas the panel showing raw measures makes the US look much more homogenous with regard to poverty. Both the percentile rankings and the absolute percentages can be important, depending on the context.

### 3.2 Validation check with county data

The 2020 county results generated in this study were identical to the CDC’s 2020 county-level estimates (Supplementary Figure 3), and these were visually similar to the pattern in the ZIP-3 SVI map for 2020 in Figure 2. The 2022, 2018, and 2016 county-level heat maps generated using the methodology in this study were visually similar but not identical to the CDC official estimates, which was expected because the 2020 CDC documentation was used to generate 2022, 2018, and 2016 SVI values (Supplementary Figure 3).

### 3.3 Demonstration of linking claims data with ZIP-3 SVI

**Figure 4:**
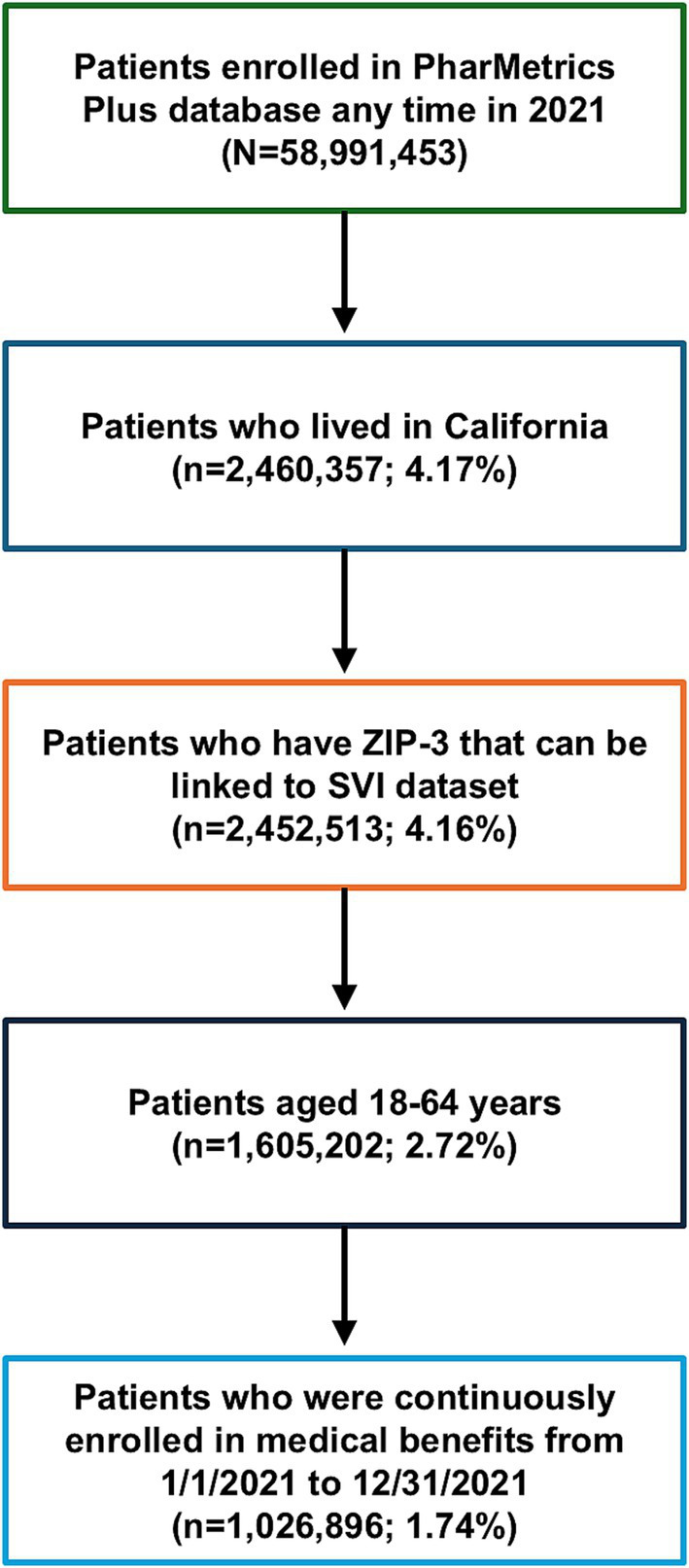
Cohort attrition for CCI and SVI analysis in patients in the PharMetrics Plus database in California for 2021. CCI, Charlson Comorbidity Index; SVI, Social Vulnerability Index; ZIP-3, 3-digit ZIP code.

**Figure 5:**
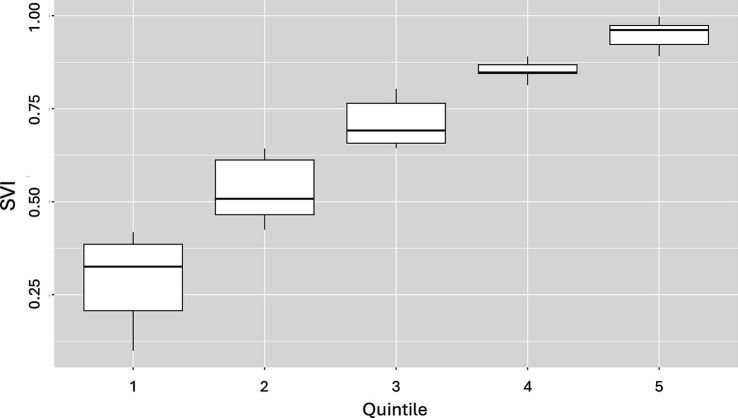
Box and whisker plots of ZIP-3 SVI scores in patients in the PharMetrics Plus database across quintiles in California for 2021^a^. SVI, Social Vulnerability Index; ZIP-3, 3-digit ZIP code. Mean SVI scores are shown above the box and whisker plots for each quintile. ^a^SVIs of the 58 ZIP-3s included in the California patient cohort were extracted and classified into quintiles relative to ZIP-3 geographic area in California, and each patient was put into 1 of the SVI quintiles.

To demonstrate how SVI can be used for health disparities research once it is linked to claims, we analyzed CCI by SVI quintiles using PharMetrics Plus health plan claims, the dataset’s patient ZIP-3 variable, and our newly created ZIP-3–level SVI variable. In PharMetrics Plus, we found 1,026,896 patients in California who were continuously enrolled in a commercial health plan in 2021 (Figure 4). In this cohort, there was roughly the same number of ZIP-3s in each quintile, but there were proportionally more patients in the lower quintiles (less vulnerable) than in the higher ones (more vulnerable) (Table 1). Box and whisker plots of SVI scores for the quintiles are shown in Figure 5.

**Table 1:** Number and percentage of ZIP-3s and patients in the PharMetrics Plus database across quintiles in California for 2021^a^.

	Number	Percentage
ZIP-3s (*n* = 58)		
SVI quintile		
1 (least vulnerable)	12	20.7
2	12	20.7
3	12	20.7
4	11	19.0
5 (most vulnerable)	11	19.0
Patients (*n* = 1,026,896)		
SVI quintile		
1 (least vulnerable)	259,867	25.3
2	171,345	16.7
3	227,803	22.2
4	197,879	19.3
5 (most vulnerable)	170,002	16.6

SVI, Social Vulnerability Index; ZIP-3, 3-digit ZIP code.

a SVIs in the 58 ZIP-3s included in the California patient cohort were extracted and classified into quintiles relative to ZIP-3 geographic area in California, and each patient was put into 1 of the SVI quintiles.

**Figure 6:**
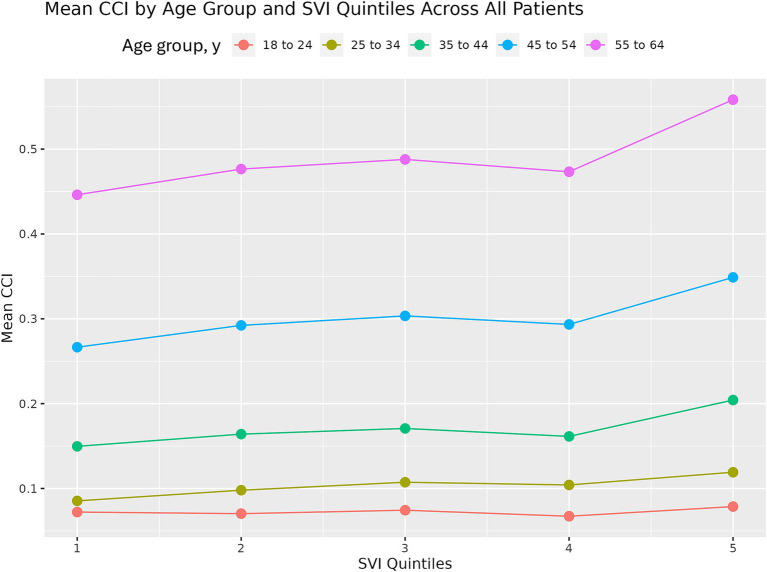
Mean CCI by age group and SVI quintile in patients in the PharMetrics Plus database in California for 2021. CCI, Charlson Comorbidity Index; SVI, Social Vulnerability Index.

Within an age group, mean CCI generally remained relatively stable (with perhaps a slight upward trend) across the first 4 quintiles (i.e., mean CCI remained stable/increased slightly as social vulnerability increased) (Figure 6). However, it appeared to spike at the highest, most vulnerable quintile. This highlights that extreme levels of vulnerability confer the greatest clinical risk. Furthermore, the difference in mean CCI between the least and most vulnerable groups increased with age. This finding suggests that the relationship between social vulnerability and patient health strengthens with increasing age. However, the absolute values of CCI were low, likely because this was not a cohort of patients with a disease of interest (e.g., those with a specific disease diagnosis) but rather a commercially insured population of working-age adults, who are likely relatively healthy. Even though ZIP-3s are relatively large geographic units and we were not looking at a specific disease cohort, we were still able to capture some differences in comorbidity burden across population quintiles, demonstrating that there are many opportunities for research and that there is a great potential for high-impact insights once the data are linked for different purposes.

## 4 Discussion

A number of health, or deprivation, indices are available in the United States to understand place-based factors’ impact on health need and health outcomes. Each measure brings its own strengths and limitations, resulting in varying levels of appropriateness, given research needs (36). For example, some measures (e.g., SVI, Social Deprivation Index, Area Deprivation Index) capture information on transportation and housing to understand physical needs in communities, and others collect information on education centers and literacy levels to understand amount of opportunity (e.g., Child Opportunity Index) (16). These measures commonly bring benefits in national and local representativeness, given their use of US Census or ACS data, which enables for census-tract– level reporting for some measures (e.g., SVI, Area Deprivation Index) (37, 38). However, not all measures are validated at less granular levels, such as US county, and most are not available at the ZIP-3 level, which is needed for integration into some datasets with patient-level data. Although the SVI is a commonly used measure in healthcare research, only having estimates for census tracts or counties limits its applications in RWD studies.

In this study, robust data on SDoH factors from the ACS, documentation on SVI methodology from the CDC, and geographic crosswalks were leveraged to construct the SVI and its components for 2016–2022 for counties, ZIP-5s, and ZIP-3s in the US. While the SVI is available from the CDC biennially at the census-tract and county level, constructing SVI for other years or levels of aggregation allows for linkage to and enrichment of patient-level data. By comparing our county-level SVI results with the county-level SVI data generated by the CDC and obtaining the same or similar results, we were able to validate that our methodology was implemented correctly. These SVI values provide an understanding of how SDoH variables compare across neighboring geographies as well as in individual geographies over time with regard to social vulnerability (39, 40). In addition to the overall SDoH metric, each individual SDoH theme or variable can be useful for disentangling specific drivers of SDoH in certain areas. Furthermore, the geographic percentiles and percentages can provide different scales for understanding the variables of interest (26).

We also demonstrated that SVI at the ZIP-3 level can be used with a large US health plan claims database, which has a variable for patient ZIP-3. If health plan claims are linked with a specific disease cohort, SVI enrichment could also be used to explore differences between patients living in more vulnerable areas vs. less vulnerable areas, such as the types of treatments prescribed, treatment adherence, and healthcare resource utilization. Crucially, this approach can be used for any kind of data that include a geographic identifier for patients. This type of analysis would allow for potential enrichment of many types of data, including insurance claims, electronic health records, registry information, and clinical trial data, enabling both patient-centered and community-oriented perspectives. Such enrichment could help identify patients at increased risk of health conditions, morbidity, or mortality or pinpoint specific areas of unmet need (23, 41).

The ability to integrate SVI with patient-level information is important because SDoH can significantly impact individuals’ health outcomes and risk factors for certain conditions (42, 43). Furthermore, some of the associations between social vulnerability and health outcomes are greater in more disadvantaged groups. For example, a study of surgical outcomes using inpatient hospital and skilled nursing facility claims covered by Medicare linked with county-level SVI from the CDC found that patients in vulnerable communities generally had worse postoperative outcomes, but the impact of social vulnerability was more pronounced in patients from racial and ethnic minority groups than in White patients, highlighting the need for both patient-level and community-level data (44).

A popular analytical method for using SVI is to stratify data by different SVI categories (e.g., quartiles, quintiles, deciles), perform the calculations for aggregate-level patient outcomes in each SVI category, and then compare across the SVI categories, as we did in our linkage demonstration. Several studies that used this aggregate-level method showed higher mortality, higher morbidity, and lower quality of life in higher social vulnerability groupings (26, 45, 46). Another usage is to regress an aggregate-level outcome on aggregate-level exposures, including SVI, and then compare the coefficients across different SVI categories. Many studies have also accounted for the spatial nature of these data through spatial distributions and weights (47–49).

At the individual level, SVI and its variables (which are area level) are often combined with individual sociodemographic and health information and used to provide insights into an individual’s susceptibility to certain health outcomes. When used to predict patient-level outcomes, SDoH and its variables are often used as confounders or effect modifiers, but there are also cases in which SDoH were used as the primary predictor of patient-level clinical outcomes (50–52).

### 4.1 Limitations of constructing and using the SVI

Several caveats should be noted for the method of SVI construction used in this study. This study used data from 2016–2022, but a similar methodology can be used for other years. However, the ACS variables would need to be validated for different years as their names change over time. Some ACS variables were missing for some geographic areas for select years, and imputation was used. However, the rate of missing values was low (~0.1%), so the choice of imputation method will likely not impact the results.

Even though ZIP-3 is the most granular geography we could potentially link with the commercial claims database, these areas are still large and heterogeneous. Because SVI (and its percentile-ranked building blocks) is distributed evenly between 0 and 1, more variation can be discerned in this range with more geographic units. While there are many more counties and ZIP-5s than ZIP-3s, the same issue potentially applies to these geographies as well. As a well-known example of this heterogeneity, 2 neighborhoods in Chicago are <10 miles apart but have a life expectancy gap of 30 years (53, 54); this heterogeneity is likely greater in a ZIP-3 that covers a much larger area. As a result, area-level SDoH and other similar predictors might have less predictive power for individual-level outcomes than for aggregate-level outcomes. Additionally, use of area-level data to guide patient-level interventions is prone to ecological fallacy (i.e., making incorrect assumptions about individuals on the basis of the profile of a group) (55, 56).

Finally, the limitations of the SVI should be noted when using this information in RWD studies. The SVI is based on the ACS, which like other national surveys, may under-report for key vulnerable populations. Additionally, questions on SDoH factors in the ACS may not collect sufficient detail to capture important differences that impact healthcare needs and access opportunities (16). Additionally, no area-level index is appropriate for all research needs. Of note, the SVI was designed to support disaster preparedness and not to capture differences in SDoH and other individual and system-level factors driving health disparities. While the SVI is frequently used and has performed well in comparative investigations on area-level indices, its limitations should be noted when attempting to draw conclusions on research findings (16).

While the approach used in this study is a step forward in evaluating health disparities, a combination of patient-level demographic and socioeconomic characteristics, in addition to SDoH, would still be the best-case scenario for health equity research.

## 5 Conclusion

Health disparities often stem from unequal access to resources and opportunities influenced by social determinants. Factors such as income, housing stability, and food security can influence the likelihood of adverse health outcomes. By integrating SDoH data into healthcare decision-making processes, organizations can work toward reducing disparities by identifying and addressing the root causes of health inequities in their patient populations. Population health management strategies aim to improve health outcomes in a group of individuals; by incorporating SDoH into population health initiatives, healthcare organizations can better understand the needs of their patient populations and develop more effective strategies for prevention, early intervention, and disease management.

At the core of these health equity goals is data, and the methodology outlined here can easily be replicated by researchers for routine enrichment of RWD. By linking SVI and its components with other sources of patient data, researchers can add area-level context and provide more robust health equity context. Being able to replicate SVI creation with a similar methodology for various years and geographic areas will allow for improved tracking of systemic barriers in health care and enhance our understanding of disparities in health outcomes by layering in the additional dimension of SDoH.

## Data Availability

The raw data supporting the conclusions of this article will be made available by the authors, without undue reservation.
