# Supplementary material for: Validating the Social Vulnerability Index for alternative geographies in the United States to explore trends in social determinants of health over time and geographic location
Source: Front Public Health. 2025 Mar 4;13:1547946. doi: 10.3389/fpubh.2025.1547946 (PMC11915720; doi:10.3389/fpubh.2025.1547946)
Supplement: Supplementary file 1 [file Data_Sheet_1.docx]

**SUPPLEMENTARY MATERIAL**

**Geographic crosswalk**

Data for 5-digit (ZIP-5) and 3-digit (ZIP-3) ZIP code geographic areas were crosswalked from the ZIP code tabulation area (ZCTA), which is the geographic unit provided by the American Community Survey. The geographic crosswalk was provided by the Health Resources and Services Administration’s Health Center Program [1]. The crosswalks from 2016 to 2022 were used for this analysis. A ZCTA was excluded if it was not in one of the 50 states or Washington, DC. If a ZCTA was classified as “populated ZCTA, missing ZIP” (i.e., the ZCTA did not correspond to a valid ZIP code), then it was excluded. The small proportion of ZCTAs that matched to multiple ZIP codes were joined to the ZIP-5 classified as “ZIP matches ZCTA,” as those classified as “spatial join to ZCTA” are typically not real, inhabited ZIPs.

**Imputation**

For every year except 2018, there were no “N/A” values for the numerators and denominators of the ZCTAs. However, there were missing numerator values in ZCTAs for 2018, specifically for variables that fell under the socioeconomic status theme (e.g., number of people living below 150% of the poverty line). An “N/A” value usually indicated that the American Community Survey 5-year estimate was significantly different (at a 90% confidence level) from the estimate for the most recent year, possibly due to a sampling issue [2]. Because the proportion of missing values for any given variable was small (~0.1%), we used single imputation based on ZCTAs within the same ZIP-3 for these missing values. Let us call the numerator variable with missing data the target variable of interest. Typically, mean imputation would directly use the mean of the target variable across the ZIP-3 to which the ZCTA belongs. However, some ZCTAs might have a small total population, and the mean of the target variable could be greater than the value of the total population, which could potentially result in a proportion of >1. Therefore, a variation of mean imputation is used:

- ZCTA: The mean of (target variable/total population) across the ZCTAs in the ZIP-3 without missing data was multiplied by the total population of the ZCTA with the missing value, and the resulting value was used as the imputed numerator

While there were no missing data for counties, a similar approach could be used, for example, for counties in a state.

**REFERENCES**

1. US Health Resources and Services Administration. Health Center Program GeoCare Navigator. <https://geocarenavigator.hrsa.gov/> [accessed June 24, 2024].

2. US Census Bureau. Notes on American Community Survey estimate and annotation values. <https://www.census.gov/data/developers/data-sets/acs-1year/notes-on-acs-estimate-and-annotation-values.html> [accessed June 19, 2024].

**Supplemental Fig. 1** Variables Used to Construct SVI (2020 CDC SVI Documentation)


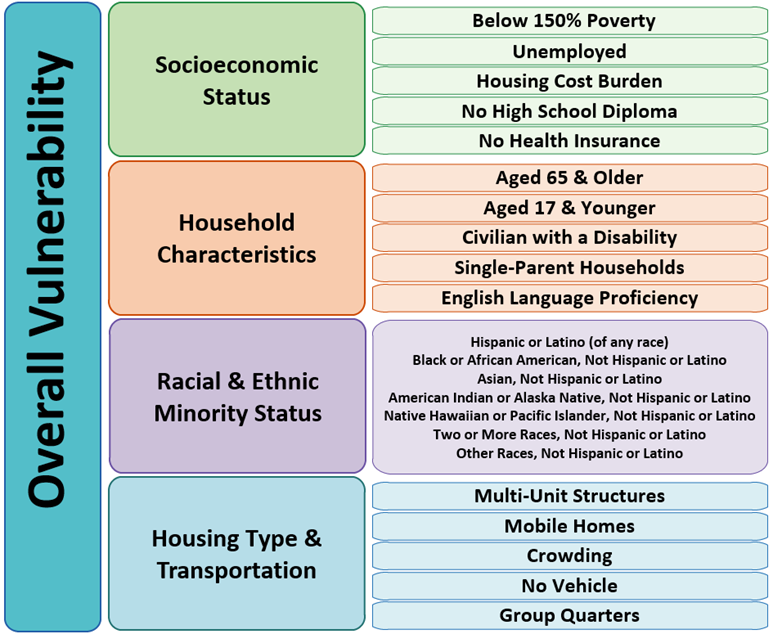


CDC, Centers for Disease Control and Prevention; SVI, Social Vulnerability Index.

This image was developed by the Agency for Toxic Substances and Disease Registry (ATSDR) and the Centers for Disease Control and Prevention (CDC). Reference to specific commercial products, manufacturers, companies, or trademarks does not constitute its endorsement or recommendation by the US Government, Department of Health and Human Services, or CDC. This image is available at the following CDC website: https://svi.cdc.gov/map25/data/docs/SVI2020Documentation_08.05.22.pdf.

**Supplemental Fig. 2** Example Workflow From Below 150% Poverty to SVI at the ZIP-3 Level


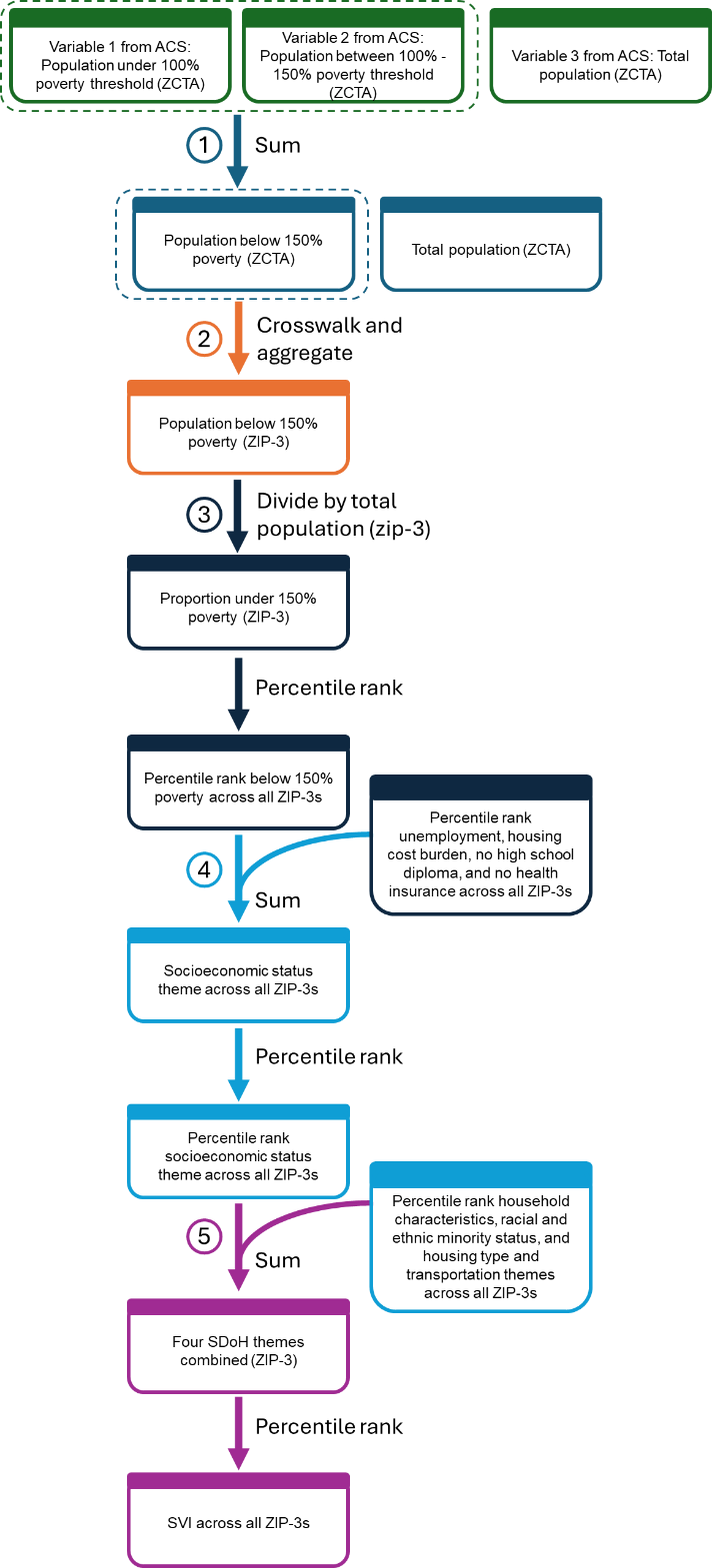


ACS, American Community Survey; SDoH, social determinants of health; SVI, Social Vulnerability Index; ZCTA, ZIP code tabulation area; ZIP-3, 3-digit ZIP code

**Supplemental Fig. 3.** Comparison of County-Level SVI Values Generated in this Study Versus Published by the CDC

**
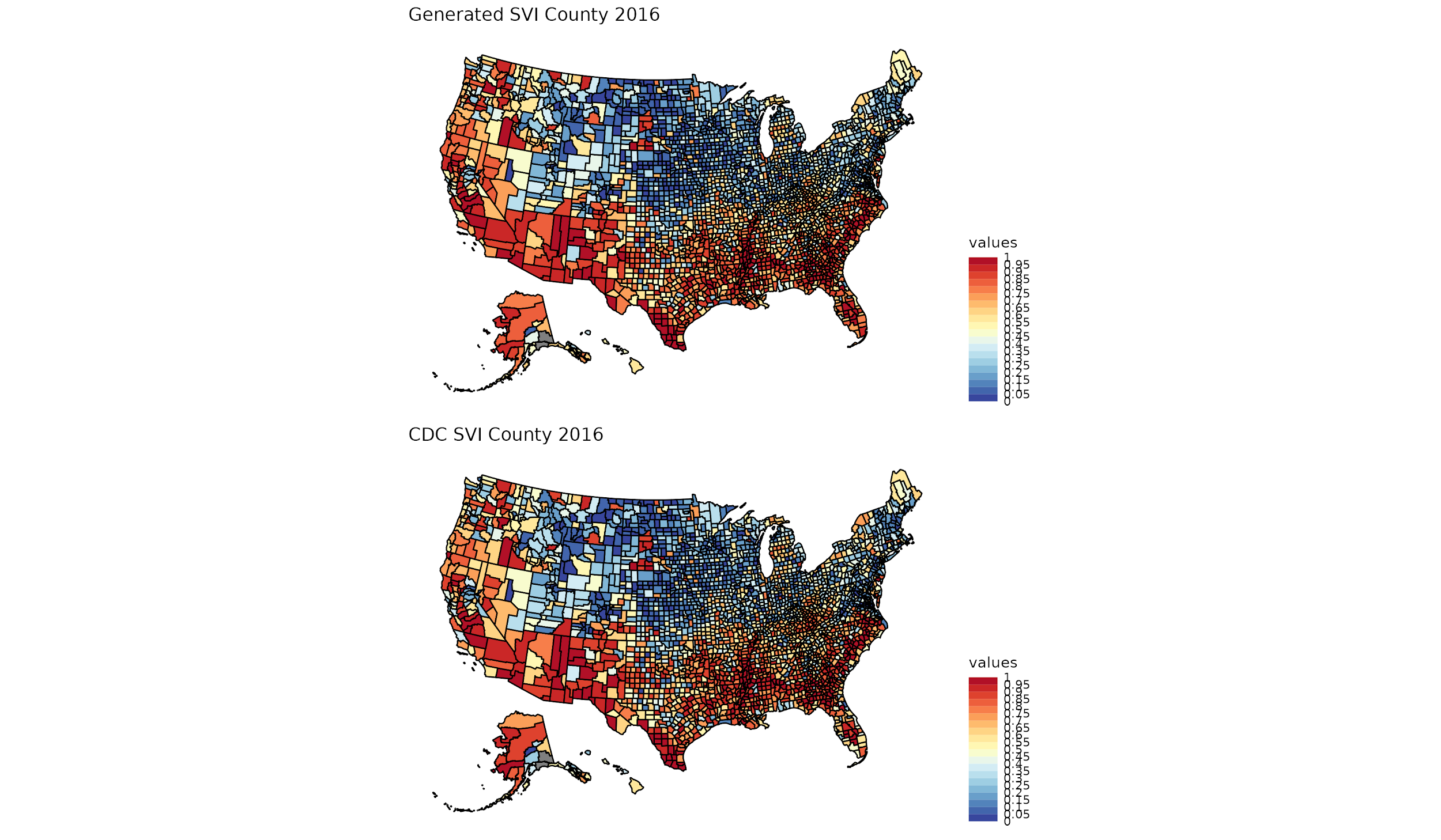

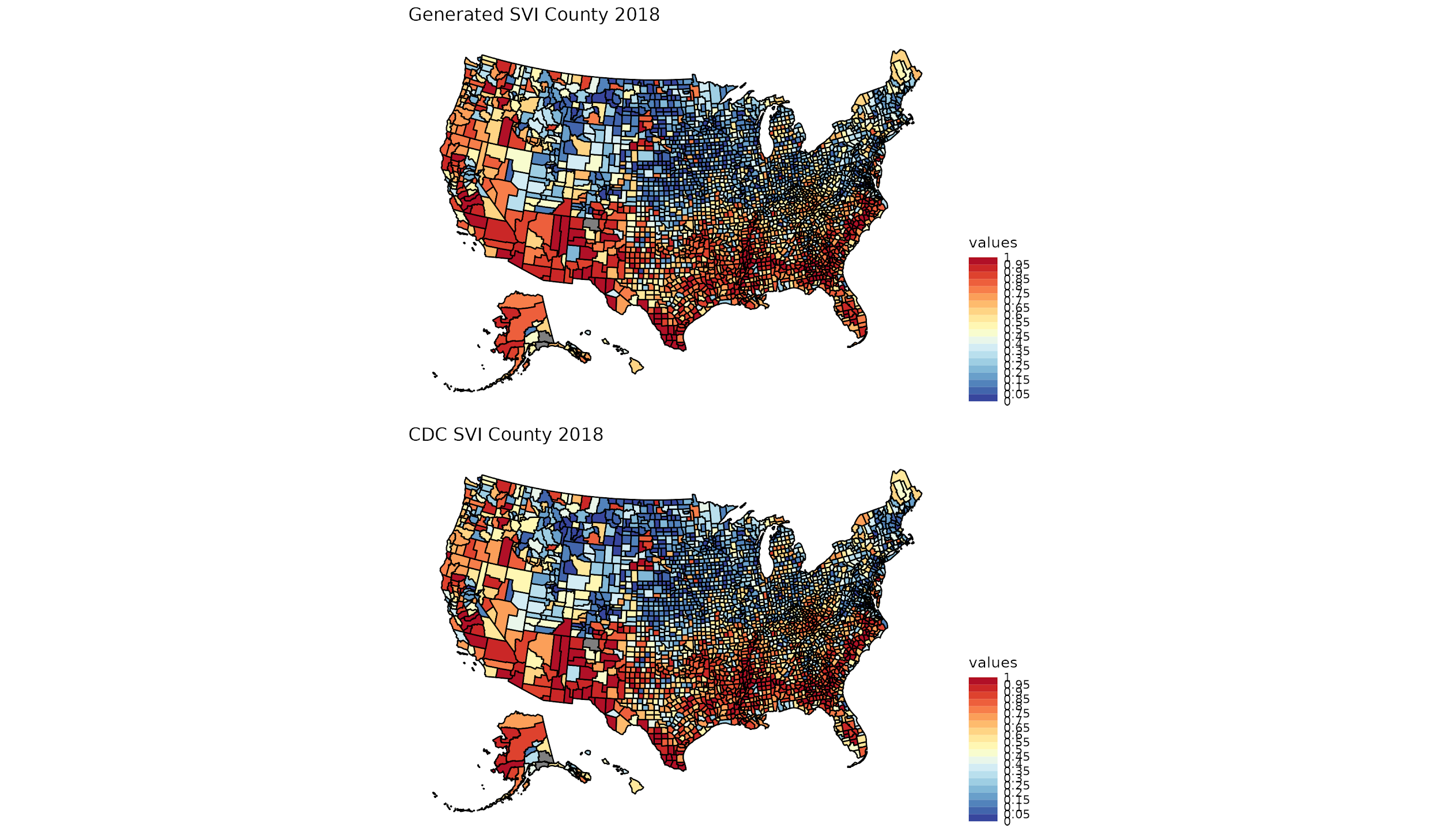

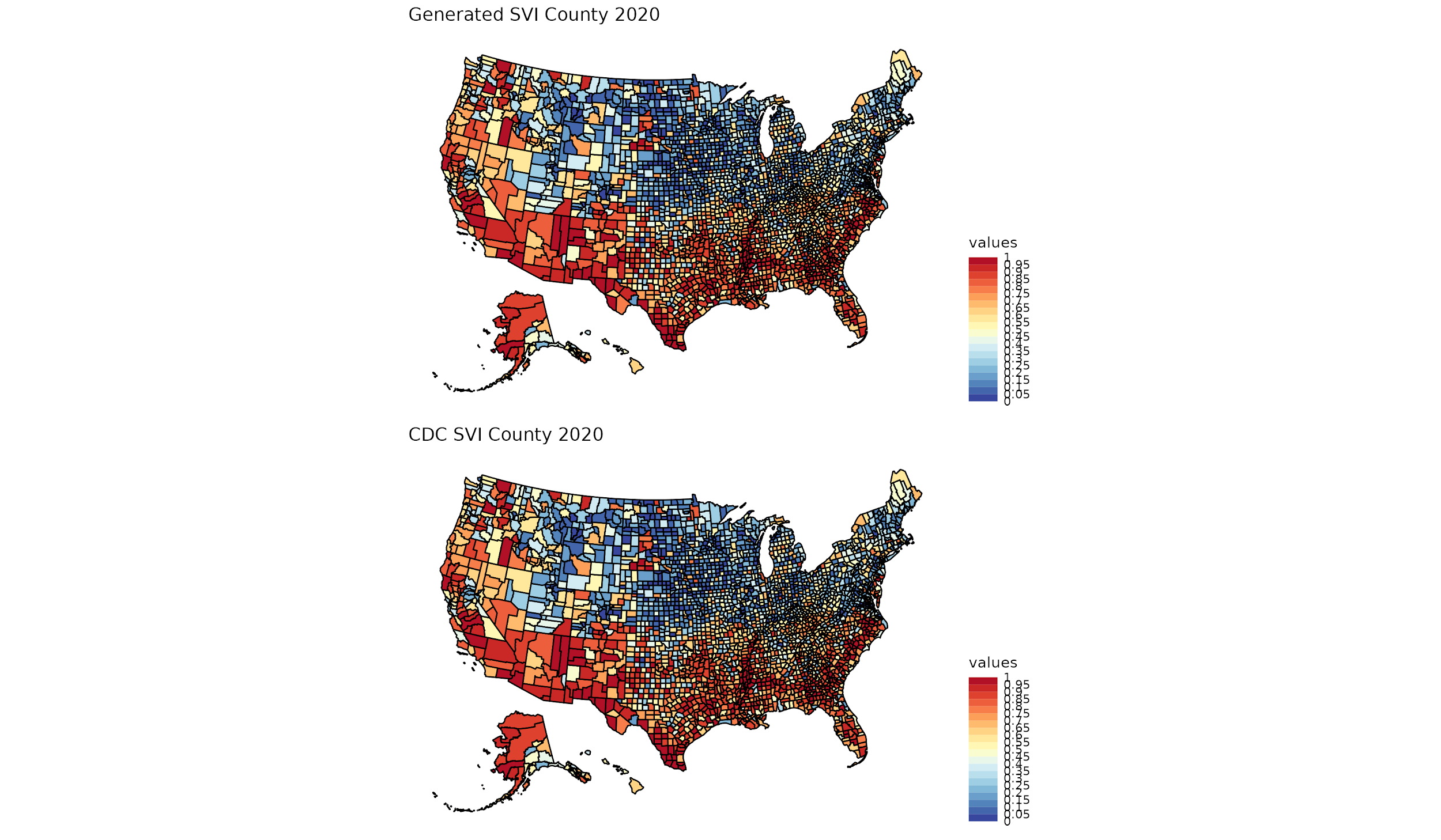

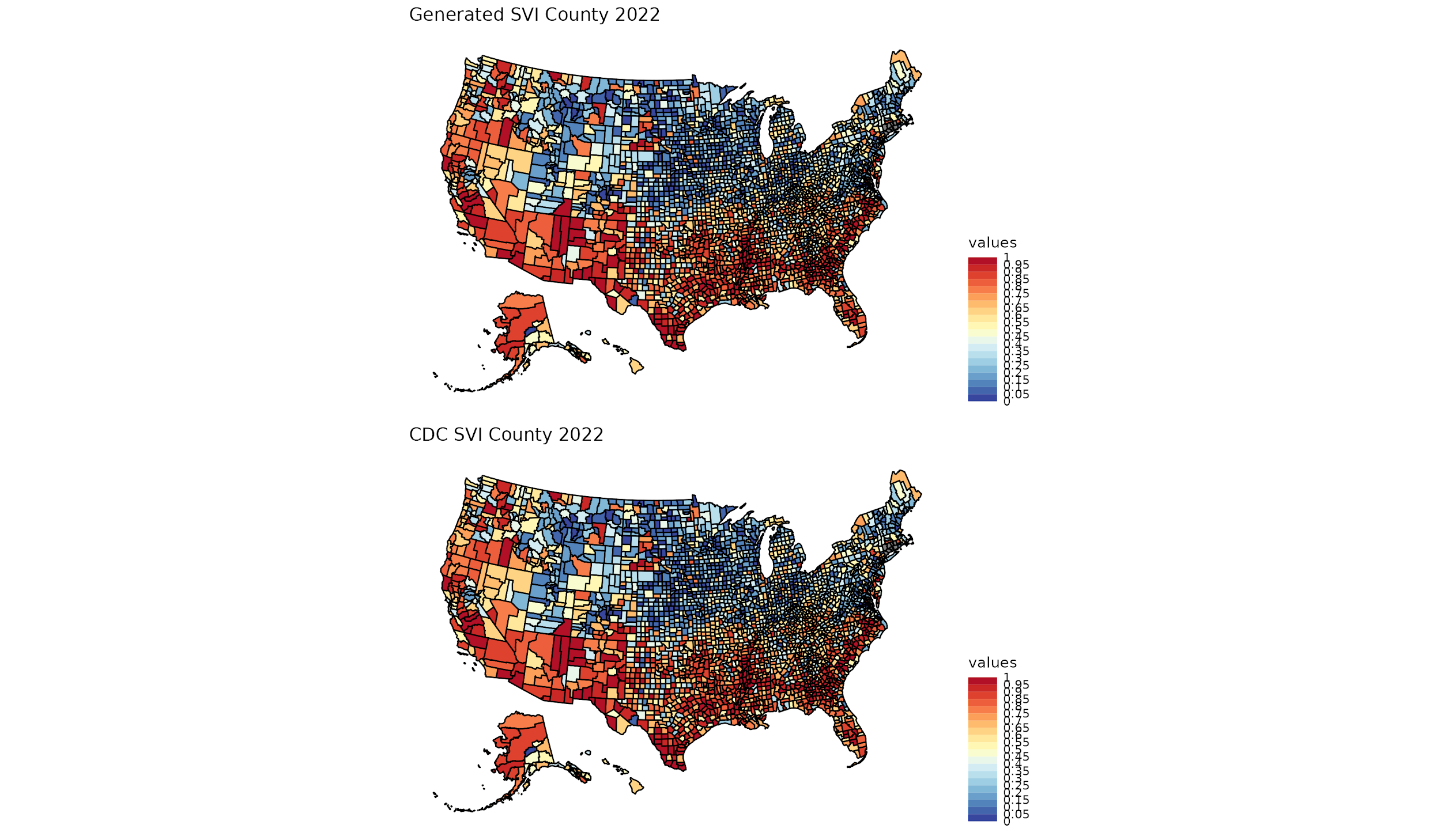
**

SVI, Social Vulnerability Index.
